# Better pain control with 8-gray single fraction palliative radiotherapy for skeletal metastases: a Bayesian network meta-analysis

**DOI:** 10.1007/s10585-020-10067-7

**Published:** 2021-02-09

**Authors:** Filippo Migliorini, Jörg Eschweiler, Andromahi Trivellas, Arne Driessen, Matthias Knobe, Markus Tingart, Nicola Maffulli

**Affiliations:** 1 Department of Orthopaedic Surgery, RWTH University Hospital Aachen, Pauwelsstraße 30, 52074 Aachen, Germany; 2 Department of Orthopaedics, David Geffen School of Medicine at UCLA, Suite 755, Los Angeles, CA 90095 USA; 3 Department of Orthopedics and Trauma Surgery, Lucerne Cantonal Hospital, Lucerne, 6000 Switzerland; 4 Department of Medicine, Surgery and Dentistry, University of Salerno, Via S. Allende, 84081 Baronissi, SA Italy; 5 School of Pharmacy and Bioengineering, Keele University School of Medicine, Thornburrow Drive, Stoke on Trent, England; 6 Barts and the London School of Medicine and Dentistry, Centre for Sports and Exercise Medicine, Queen Mary University of London, Mile End Hospital, 275 Bancroft Road, London, E1 4DG England

**Keywords:** Radiotherapy, Bone metastases, Pain, Fracture, Survivorship

## Abstract

External Beam Radiotherapy (EBRT) allows remarkable pain control in patients with skeletal metastases. We performed a Bayesian network meta-analysis comparing the most commonly used radiotherapy regimens for palliative management in patients with skeletal metastases. The main online databases were accessed in October 2020. All randomized clinical trials evaluating the irradiation of painful bone metastases were considered. The following irradiation patterns were analysed and included in the present network meta-analysis: 8 Gy- and 10 Gy/single fraction, 20 Gy/5 fractions, 30 Gy/10 fractions. The Bayesian hierarchical random-effect model analysis was adopted in all comparisons. The Log Odds-Ratio (LOR) statistical method for dichotomic data was adopted for analysis. Data from 3595 patients were analysed. The mean follow-up was 9.5 (1 to 28) months. The cumulative mean age was 63.3 ± 2.9. 40.61% (1461 of 3595 patients) were female. The 8Gy/single fraction protocol detected reduced rate of “no pain response” (LOR 3.39), greater rate of “pain response” (LOR-5.88) and complete pain remission (LOR-7.05) compared to the other dose patterns. The 8Gy group detected a lower rate of pathological fractures (LOR 1.16), spinal cord compression (LOR 1.31) and re-irradiation (LOR 2.97) compared to the other dose patterns. Palliative 8Gy/single fraction radiotherapy for skeletal metastases shows outstanding results in terms of pain control, re-irradiations, pathological fractures and spinal cord compression, with no differences in terms of survivorship compared to the other multiple dose patterns.

**Level of evidence**: I, Bayesian network meta-analysis of RCTs.

## Introduction

Metastatic disease is a common source of bone pain [1]. Bone metastases are debilitating and lead to pain, impaired mobility, malignant hypercalcemia, pathological fractures, and, when the spinal cord is involved, neurological disability [2]. Also, severe hypercalcemia can lead to cardiac and kidney failure [3], with marked decrease in median survival [4]. As lung, thyroid, and renal cancer, multiple myeloma and melanoma often metastasize to bone [5, 6], bone metastases are common in the spine, pelvis and hip, shoulder, and distal femur [7] with elbow and knee metastases typical of lung cancer metastases [8]. The exact incidence of bone metastases is still unknown [3], but they impact greatly on patients and health care systems [9]. Approximately 70% to 90% of the patients who died from breast or prostate cancer develop bone metastases [10, 11]. Over the past several years, an increased interest on External beam radiotherapy (EBRT) as management for selected patients with skeletal metastases has emerged [12]. EBRT achieved satisfactory results in pain control along with reduced burden in terms of both hospital attendances and side effects [13–15]. The dose pattern is measured in Gray (Gy), while the number of sessions is called fractions. Several studies have shown that 30 Gy in 10 fractions, 20 Gy in 5 fractions, or 8 Gy in a single fraction afford optimal pain control with acceptable adverse effects [16]. Given the complexity and lack of consensus concerning the palliative radiotherapy for patients with skeletal metastases, we performed a Bayesian network meta-analysis comparing the three most commonly used dose patterns: 8Gy in one fraction, 20Gy and 30Gy in five and ten fractions, respectively.

## Material and methods

### Search strategy

The present Bayesian network meta-analysis was performed according to the PRISMA extension statement for reporting of systematic reviews incorporating network meta-analysis of health care interventions [17]. To guide the search, a preliminary protocol was performed:

- P (population): skeletal metastases;
- I (intervention): palliative radiotherapy;
- C (comparison): 8Gy *vs* 20Gy *vs* 30Gy;
- O (outcomes): pain scores and medications, further therapies, side effects, survivorship.

### Literature search

Two authors (FM; JE) independently performed the literature search in October 2020. The databases accessed were Pubmed, Google Scholar, Scopus. The following keywords were used alone and in combination: metastasis, skeletal, bone, radiotherapy, irradiation, Gray, survivorship, pain, treatment, fractures, spinal cord compression, cancer, 8Gy, 20Gy, 30Gy. The same authors independently screened the resulting articles. If title and subsequent abstract matched the topic, the full-text article was accessed. The bibliographies of the studies of interest were also reviewed by hand.

### Eligibility criteria

All randomized clinical trials evaluating irradiation of painful bone metastases were considered for analysis. Only articles of level I evidence according to the Oxford Centre of Evidenced-Based Medicine [18] were included in the present study. Given the authors languages capabilities, articles in English, German, Italian, Portuguese and Spanish were considered eligible. Letters to editors, expert opinions, case series, and other review articles were not considered for analysis. Cadaveric, animal, in vitro or computational study were excluded. The following irradiation patterns were analysed and included in the present network meta-analysis: 8Gy and 10Gy in one fraction, 20Gy in five fractions, 30GY in 10 fractions. Articles reporting data concerning different irradiation patterns were excluded. Only articles providing quantitative data under the outcomes of interest were included. Missing data under the outcomes of interest warranted the exclusion from the present work. Disagreements between the authors were mutually debated and solved.

### Outcomes of interest

Two authors (FM; JE) independently extracted data from the included articles. The following variables were collected: author and year of publication, follow-up duration, number of treated patients and respective mean age and gender. Furthermore, the inclusion and exclusion criteria were reported along with the site of the primary tumour and localization of metastases. Concerning the treatment, we collected data regarding irradiation site and doses, pre- and post-treatment pain scores and medication, further therapies, side effects and survivorship.

### Statistical analysis

The statistical analysis was performed by the main author (FM). To analyse patient baseline comparability, the analysis of variance (ANOVA) test was performed, with values of P > 0.1 considered satisfactory. The endpoint pain medication consumption relates to the percentage of patients using that drug. For analytical statistics, the STATA Software/MP (STATACorp, College Station, TX) was used. The Bayesian hierarchical random-effect model analysis was adopted in all comparisons. A reference value was set in all comparisons. The Log Odds-Ratio (LOR) and standard error (SE) statistical method for dichotomic data was adopted for analysis. The edge plot was performed to evaluate contribution of direct and indirect comparisons among the networks. To verify transitivity among studies, the overall inconsistency was evaluated through the equation for global linearity via the Wald test. Values of P > 0.05 could not reject the null hypothesis, and the consistency assumption could be accepted at the overall level of each treatment. The overall estimated effect of the comparisons and ranking for each endpoint was evaluated through the interval plot. Both the confidence interval (CI) and percentile interval (PrI) was set at 95%. Funnel plots were performed to evaluate the risk of publication bias among the studies.

## Results

### Literature search

**Fig. 1:**
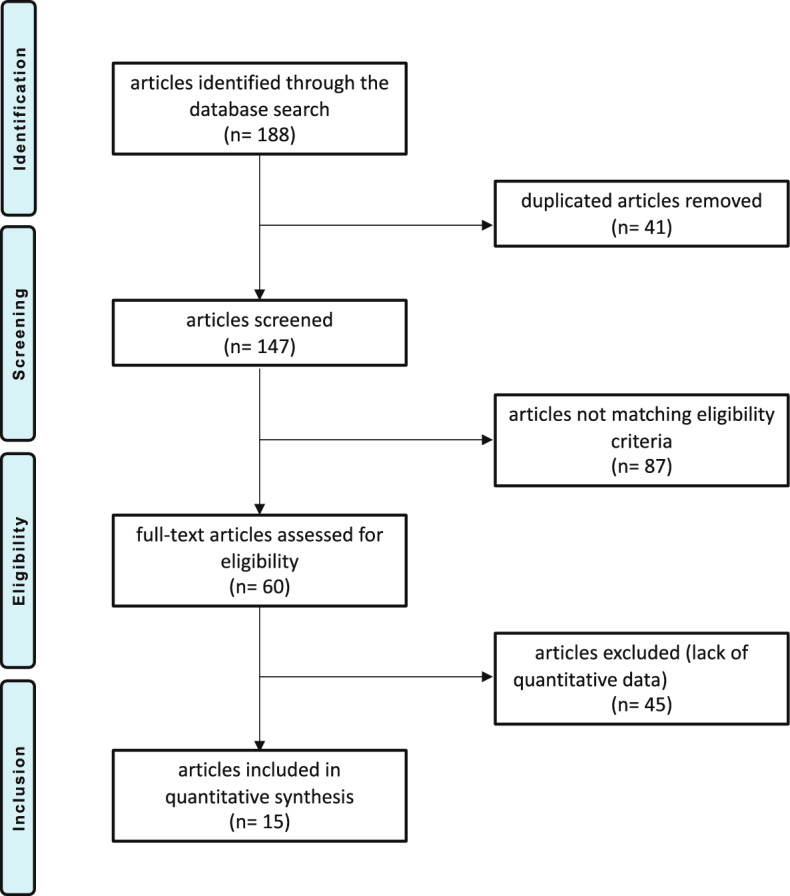
Flow-chart of the literature search

The literature search resulted in 188 publications, of which 41 were duplicates. A further 82 articles were excluded because not matching the eligibility criteria: not randomized trial or poor level of evidence (45); concerning different doses or fractionations and radiotherapeutic protocols (23); language limitation (1), uncertain results (3), other (10). Another 45 articles were rejected for not reporting quantitative data under the outcomes of interest. Finally, 15 randomized clinical trials were eligible for analysis in the present study (Fig. 1).

### Study demographics

In the present Bayesian network meta-analysis, data from 3595 patients were analysed. The mean follow-up was 9.5 (1 to 28) months. The cumulative mean age was 63.3 ± 2.9. 40.61% (1461 of 3595 patients) were female. The ANOVA test evidenced no differences regarding age or sex of patients among the studies at baseline (P > 0.5). Demographic data are shown in Table 1.

**Table 1:** Demographic data of studies included for analysis

Author, year	Follow-up (months)	Dose	Patients (n)	Mean age	Female gender (%)	Inclusion criteria	Exclusion criteria	Primary tumour site	Metastases localization/irradiated sites
Amouzegar-Hashemi et al. 2008 [44]	1	8 Gy/1 fraction	27	51.6		(1) adult (2) uncomplicated metastases	(1) spinal cord compression (2) existing or impending pathologic fracture		
		30 Gy/10 fractions	31	51.6					
Bone Pain Trial Working Party, 1999 [45]	12	8 Gy/1 fraction	382	67.0	50	(1) age > 18	(1) pathological fracture of a long bone (2) previous radiotherapy to the index site	36% Breast, 33% Prostate, 13% Lung, 2% Unknown, 16% Other	27% Pelvis/hip, 20% Spine, 11% Ribs, 10% Thoracic spine, 6% Femur, 3% Lumbar Spine, 2% Cervical Spine, 21% Other
		30 Gy/10 fractions	8	66.0	46			36% Breast, 36% Prostate, 10% Lung, 3% Unknown, 15% Other	29% Pelvis/hip, 20% Spine, 10% Ribs, 8% Thoracic Spine, 6% Femur, 3% Lumbar Spine, 2% Cervical Spine, 22% Other
		20 Gy/5 frarctions	370						
Foro Arnalot et al. 2008 [46]	3	8 Gy/1 fraction	78	64.8	42	(1) age > 18 (2) life expectancy > 1 month	(1) pathological fracture or impending fracture according to Mirels’ criteria (2) evidence of spinal cord compression (3) multiple metastasis (4) prior radiotherapy at the same site (5) impossible pain assessment due to an overall poor healthy state or to difficulties in applying the ordinal pain scale	27% Breast, 24% Lung, 24% Prostate, 4% Myeloma, 13% Digestive, 8% Others	45% Pelvis, 37% Spine, 10% Long bone, 8% Others
		30 Gy/10 fractions	82	63.4	43			27 % Breast, 27% Lung, 26% Prostate, 11% Myeloma, 7% Digestive, 2% Others	34% Pelvis, 36% Spine, 18% Long bone, 12% Others
Gutierrez Bayard et al. 2014 [47]	12	8 G**y**/1 fraction	45	62.6	38	(1) Karnofsky Performance > 50 (2) life expectancy > 6 weeks	(1) large bony lesions on the spine or pelvis requiring fixation (2) impending or established pathological fracture (3) spinal cord compression (4) previous radiotherapy at the same site	11% Lung, 31% Prostate, 40% Breast, 18% Other	16% Limb, 62% Axial Skeletal, 7% Cranial, 13% Axial + Limbs, 2% Limbs + Cranial
		30 Gy/10 fractions	45	61.8	42			20% Lung, 38% Prostate, 20% Breast, 22% Other	7% Limb, 73% Axial Skeletal, 7% Cranial, 7% Axial + Limbs, 9% Limbs + Cranial, 2% Axial + Cranial
Hartsell et al. 2005 [48]	3	8 Gy/1 fraction	445	65.5	51	(1) age > 18 (2) diagnosis of malignancy of breast and prostate (3) Karnofsky Performance Status > 40 (4) life expectancy > 3 months (5) Brief Pain Inventory > 5/10 (6) more than 3 separate metastases (7) no introduction of any systemic therapy < 30 days	(1) pathological or impending fracture (2) planned surgical fixation (3) spinal cord or cauda equina compression		56% Weight bearing, 44% Non-weight bearing
		30 Gy/10 fractions	443	65.1	50				56% Weight bearing, 44% Non-weight bearing
Kaasa et al. 2006 [49]	18	8 Gy/1 fraction	186	66.7	40	(1) Karnofsky Performance Status > 40 (2) life expectancy > 6 weeks	(1) previous irradiation (2) spinal cord compression (3) planned orthopaedic surgery, unable to complete the QOL assessment tools, life expectancy less than 6 weeks	11% Lung, 38% Prostate, 28% Breast, 23% other	39% Spine, 35% Pelvis, 20% Extremities, 5% Other
		30 Gy/10 fractions	190	67.0	48			11% Lung, 36% Prostate, 32% Breast, 22% Other	37% Spine, 35% Pelvis, 19% Extremities, 9% Other
Kagei et al. 1990 [50]	2	8 Gy/1 fraction					(1) chemotherapy on same day as radiotherapy (2) fracture determining spine compression (3) pathological fracture		
		10 Gy/1 fraction							
		15 Gy/1 fraction							
		20 Gy/4 fractions							
		25 Gy/5 fractions							
		30 Gy/6 fractions							
Kirkbride et al. 2000 [51]	3	8 Gy/1 fraction	137						
		20 Gy/5 fractions	124						
Koswig et al. 1999^52^]	6	8 Gy/1 fraction				(1) diagnosis of breast, lung, prostate and kidney carcinoma (2) solitary osteolysis with or without fracture risk (3) osteolytic lesion suitable for bone density measurements via CT	(1) prior irradiation (2) new systematic therapies < 2 weeks		
		30 Gy/10 fractions							
Loblaw et al. 2006 [53]		8 Gy/1 fraction	23	66.5		(3) age > 18 (2) life expectancy > 4 months	(1) multiple myeloma (2) changes in systemic therapy for their cancer < 1 month		
		20 Gy/5 fractions	21	66.5					
Majumder et al. 2012 [54]		8 Gy/1 fraction	31	60.0	19.4	(1) age < 75 years (2) Karnofsky Performance Status > 40	(1) spinal cord compression (2) pathologic fracture (3) wide area of multiple spinal metastases (4) existing bone disease (5) previous radiation to spine or any site overlapping the treatment site	77% Prostate, 4% Breast, 2% Lung	3 % Cervical, 64.5% Lumbar, 6.5% Sacral, 26% Thoracic
		30 Gy/10 fractions	33	58.0	15			8% Prostate, 1% Cervix, 3% Lung, 1% Breast	6 % Cervical, 54% Lumbar, 9% Sacral, 30% Thoracic
Nielsena et al. 1998 [55]	5	8 Gy/1 fraction	120	67.0	44	(1) life expectancy more than 6 weeks	(1) previous radiotherapy in the same site (2) pathological fractures (3) spinal cord compression (4) changes in the systemic treatment both before or after the radiation	35% Breast, 38% Prostate, 13% Lung, 14% Other	47% Dorsal/Lumbar Spine, 20% Pelvis, 15% Hip/Femur, 18% Other
		20 Gy/4 fractions	119	67.0	60			44% Breast, 29% Prostate, 13% Lung, 14% Other	37%Dorsal/Lumbar Spine, 22% Pelvis, 22% Hip/Femur, 19% Other
Price et al. 1986 [56]	28	8 Gy/1 fraction	108	61.4		(1) life expectancy > 6 weeks	(1) pathological fractures of long bone (2) previous radiotherapy in the same site (2) change in systemic therapy < 6 weeks	36% Breast, 21% Lung, 7% Prostate, 2% Kidney, 6% Myeloma, 28% others	1% Cervical Spine, 51% Dorsal/Lumbosacral Spine, 22% Pelvis/Hip, 8% ribs, 17% Others
		30 Gy/10 fractions	95	62.0				39% Breast, 19% Lung, 10% Prostate, 3% Kidney, 1% Myeloma, 20% Others	2% cervical spine, 47% Dorsal/Lumbosacral Spine, 30% Pelvis/Hip, 5% Ribs, 16% Others
Roos et al. 2005 [57]	12	8 Gy/1 fraction	137	67.0	27	(1) pain or dysaesthesia predominantly of a neuropathic nature (2) life expectancy > 6 weeks	(1) neuropathic pain distribution (2) prior radiotherapy in the same site (3) spinal cord compression (4) cauda equina syndrome (5) pathological fracture of long bone (6) change in systemic therapy < 6 weeks	7% Breast, 33% Lung, 28% Prostate, 33% Others	85% Spine, 12% Ribs, 2% Other
		20 Gy/5 fractions	135	68.0	29			10% Breast, 29% Lung, 30% Prostate, 30% Others	92% Spine, 6% Ribs, 2% Other
Sande et al. 2009 [58]	7	8 Gy/1 fraction	85	67.2	39	(1) Karnofsky Performance Status > 40 (2) life expectancy > 6 weeks	(1) previous irradiation in the same site (2) spinal cord compression (3) bone surgery	33% Prostate, 21% Breast, 14% Lung, 26% Other, 7% Unknown	9% Upper limbs, 13% Lower limb, 5% Cervical Spine, 14% Thoracic Spine, 16% Lumbar Spine, 2%Sacral Spine, 7% Thorax, 29% Pelvis
		30 Gy/10 fractions	95	65.2	48			27% Prostata, 29% Breast, 14% Lung, 25% Other, 3% Unknown	11% Upper Limb, 13% Lower Limb, 6% Cervical Spine, 15% Thoracic Spine, 10% Lumbar Spine, 13% Thorax, 23% Pelvis

### Pain medication consumption and survivorship

Pain medication consumption was not analysed in the 20Gy group because of lack of quantitative data. Administration of pain medication was notably reduced post-treatment in the 8Gy and 30Gy groups. No administration of pain medication improved to + 19.50% and + 21.83% in the 8Gy and 30Gy groups, respectively, while the use of nonsteroidal anti-inflammatory drugs (NSAID) was reduced to − 13.92% and − 8.65%. Overall consumption of weak narcotics was reduced to − 3.33% and − 8.50% in the 8Gy and 30Gy groups, respectively. The intake of strong narcotics was reduced to − 18.25% in the 8Gy group, while in the 30Gy group an increase to +3.80% was detected during last follow-up. The mean survival was 7.93 ± 1.87 months in the 8Gy group, 6.65 ± 2.62 months in the 20Gy group, and 8.71 ± 0.8 months in the 30Gy treatment group. Pain medication consumption and survivorship are shown in Table 2.

**Table 2:** Pre- and post-treatment medication assumption and survivorship

Dose	Patients (*n*)	Mean survivorship (*months*)	Pain medication assumption pre-therapy (%)				Pain medication assumption post-therapy (%)				Improvement (%)			
			No use	NSAID	Weak narcotics	Strong narcotics	No use	NSAID	Weak narcotics	Strong narcotics	No use	NSAID	Weak narcotics	Strong narcotics
8 Gy/1 fraction	1804	7.93 ± 1.9	7.50 ± 2.6	37.25 ± 20.6	24.33 ± 11.9	48.50 ± 24.3	27.00 ± 8.2	23.33 ± 10.8	21.00 ± 5.9	30.25 ± 27.6	+ 19.50	- 13.92	- 3.33	- 18.25
20 Gy/5 fractions	769	6.65 ± 2.6	6.00 ± 2.8	49.50 ± 29.0	9.00 ± 3.7	67.00 ± 18.4								
30 Gy/10 fractions	1022	8.71 ± 0.8	9.50 ± 3.5	31.00 ± 17.0	29.50 ± 2.1	30.00 ± 11.3	31.33 ± 8.1	22.35 ± 14.3	21.00 ± 7.4	33.80 ± 25.2	+ 21.83	- 8.65	- 8.50	+ 3.80

### Network comparisons

**Fig. 2:**
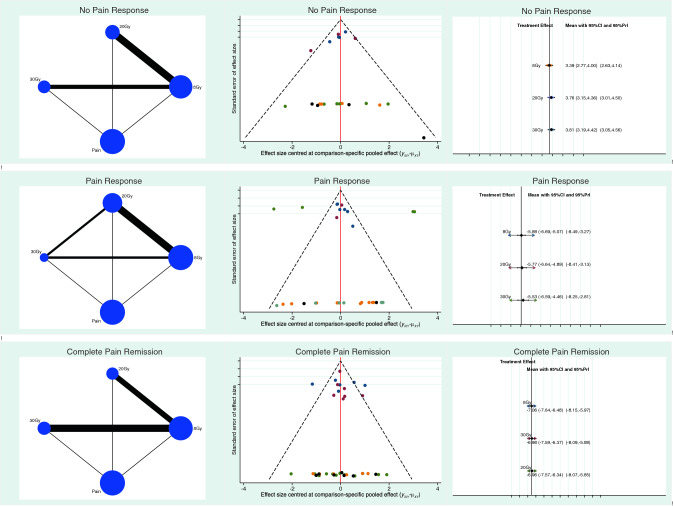
Results of the network comparisons concerning pain control

The endpoint “no pain response” scored lower in the 8Gy group (LOR 3.39; SE 0.32; 95% CI 2.77 to 4.00; 95% PrI 2.63 to 4.14). The endpoint “complete pain remission” scored better in the 8Gy group (LOR -7.05; SE 0.29; 95% CI − 7.64 to − 6.47; 95% PrI − 8.14 to − 5.97). The 8Gy group demonstrated a better pain response to the therapy (LOR -5.88; SE 0.41; 95% CI − 6.69 to − 5.07; 95% PrI − 8.49 to − 3.26). The equation for global linearity via the Wald test detected no statically significant inconsistency (P > 0.5). Results of the network comparisons concerning pain control are shown in Fig. 2.

**Fig. 3:**
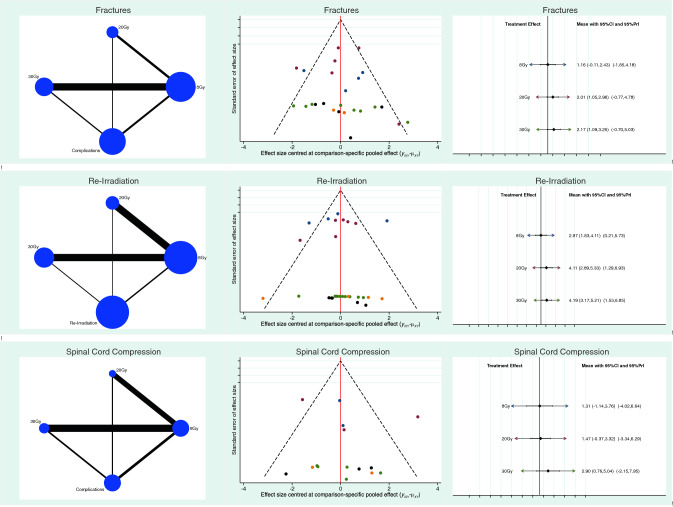
Results of the network comparisons concerning complications.

The 8Gy group showed a lower rate of pathological fractures compared to the other groups (LOR 1.16; SE 0.65; 95% CI -0.11 to 2.43; 95% PrI -1.85 to 4.18). In the 8Gy group there was a significant lower rate of spinal cord compression compared to the other groups (LOR 1.31; SE 1.25; 95% CI − 1.14 to 3.76; 95% PrI − 4.02 to 6.64). In terms of reduced re-irradiation, the the 8Gy group detected reduced rate compared to the other cohorts (LOR 2.97; SE 0.58; 95% CI 1.83 to 4.11; 95% PrI 0.21 to 5.73). The equation for global linearity via the Wald test detected no statically significant inconsistency (P > 0.5). Results of the network comparisons concerning complications are shown in Fig. 3.

## Discussion

This Bayesian network meta-analysis showed that a single dose of 8Gy/one fraction radiation therapy is superior to multiple doses (20Gy and 30Gy) in terms of palliative control of pain in patients with skeletal metastases. Further, the 8Gy/one fraction protocol showed lower rate re-irradiation and complications, such as spinal cord compression and fractures. No differences in survivorship between the different dose patterns was detected.

The palliative 8Gy radiotherapy group performed better overall. Administration of pain medication was significantly lower in the 8Gy group compared to the 30Gy group. The network analysis reported a statistically significant lower risk of pathological fracture and spinal cord compression in the 8Gy group compared to the 20Gy and 30Gy groups. Partial and overall pain recurrence were remarkably lower in the 8Gy group compared to both 20Gy and 30Gy groups. Furthermore, the 8Gy group reported the lowest rate of non-response among patients. Concerning the endpoint of re-irradiation, no statistical significance was detected between the regimens studied in the present report. Of note, one single fractionation of radiation offers greater patient and caregiver convenience.

The *seed-and-soil* theory hypothesize that, given several cytokines and growth factors, bone tissue (the *soil*) can provide an optimal field for the metastases (the *seed*) [19]. The process that leads to metastatic growth is regulated by a signalling pathway between the microenvironment and tumoral cells [20, 21]. This signalling induces growth of the tumour by interacting with the bone homeostasis. The first studies on tumour genesis demonstrated a “vicious cycle” between bone and tumour cells [21]. Tumour cells secrete cytokines, such as parathyroid hormone related protein (PTHrP), stimulating the release of RANKL from osteoblasts, promoting osteoclast bone resorption [11, 22]. Bone resorption then releases growth factors, in turn promoting tumour growth, and the cycle continues. Modern anti-tumoral therapies are based on this rationale to directly inhibit osteoclasts [23]. However, some anti-tumour therapies such as hormone deprivation (anti-androgen or anti-oestrogen) or corticosteroids exert a negative impact on bone quality, resulting in osteoporosis [24]. Bisphosphonates, such as zoledronate, bind hydroxyapatite and interact with osteoclasts, promoting apoptosis, thus inhibiting bone resorption [25]. Bisphosphonates delay the advance of existing bone metastasis and reduce the risk of developing new bony lesions in patients with breast cancer and multiple myeloma [26, 27]. Bisphosphonates also reduce skeletal complications in hormone-refractory prostate cancer [28], non-small cell lung cancer, and other urologic malignancies [29, 30]. Their use is recommended in patients affected by breast cancer with signs of bone metastasis [31]. Denosumab is a monoclonal antibody that inhibits RANKL activity, a ligand in the signalling pathway that regulates osteoclast differentiation and activation. Thus, there is widespread interest in it for bone metastasis treatment, particularly when zoledronate is no longer effective [32]. Furthermore, Denosumab does not accumulate in bone, allowing quick reversal after its suspension [32]. However, the considerably increased risk of jaw osteonecrosis represents a major complication of bisphosphonates [33]. External beam radiotherapy (EBRT) is a common alternative treatment options for pain in uncomplicated bone metastases [16]. Chow et al. [13] reported partial pain relief in 50% to 80% of patients with bone metastasis receiving EBRT. Furthermore, complete relief from metastatic bone pain was reported by almost 30% of patients [13]. Interestingly, the cause of pain in bone metastases is still unknown [34]. Radiation destroys tumour cells, promoting bone reparation, but the rapid pain relief poses a question on the true source of bone pain. Hoskin et al. [35] found a possible explanation of the reported pain relief after radiotherapy, hypothesising that the source of pain is intrinsic to the bone (osteoclasts) rather than the tumour. This partially explains the pain relief observed even with bisphosphonate treatment, as they directly act on the osteoclasts.

Regarding surgical intervention, an impending or frank pathological fracture from bone metastases is a classical indication for internal fixation. Surgery is also indicated when metastases cause spinal cord involvement or peripheral nerve compression. Two retrospective cohort studies evaluated the outcome of prophylactic surgical fixation of impending fractures [36, 37]. Fixation of impending fractures leads to a reduction of total blood loss, shorter hospitalization, improved function, and longer survival compared to surgical fixation of pathological fractures [36, 37]. The Mirels score evaluates the potential necessity for prophylactic surgery [38]. Another way to evaluate the potential need for prophylactic surgery is the assessment of bone with CT scan [39]. This technique compares the structural rigidity of the bone matrix of the contralateral side and has shown superior sensitivity and specificity compared to Mirels criteria [39]. Surgical outcomes and survival, however, depend strictly on the preoperative health of a patient [40]. The Goldman classification is useful to evaluate patient pre-operative health status [41]. This score assesses the surgical risk based on cardiac, respiratory, and other secondary factors. Analysing death prognostic factors, Nathan et al. [42] found that primary diagnosis, use of systemic therapy, Eastern Cooperative Oncology Group (ECOG) performance status, number of bone metastases, presence of visceral metastases, and serum haemoglobin, albumin, and lymphocyte counts were significant in predicting survival.

Major points of strength of this Bayesian network meta-analysis are the comprehensive nature of the literature search and the strict eligibility criteria for study inclusion. Several different palliative fractionation schedules and doses (e.g. 12Gy, 15Gy, 16Gy in one fraction, 22.5Gy in 5 fractions, 24Gy in 6 fractions, 40Gy in 20 fractions, and many other) have been described [43]. Lack of data or low level of evidence, however, did not allow inclusion of some studies in the present meta-analysis. Thereby, only the most commonly used dose patterns were compared. The small number of RCTs included in this study represents the most important limitation. Furthermore, this reflects the lack of evidence and consensus on this topic. Further high-quality studies are required to improve our current understanding in this field. Other important limitations are the lack of data and the heterogeneous eligibility criteria among the studies included. This increases publication bias, which negatively influences our recommendation. Given the lack of quantitative data, it was not possible to analyse the different anatomical districs (spine, lower and upper limb) separately. Given these limitations, data from this study must be interpreted with caution.

## Conclusion

Palliative 8Gy/single fraction radiotherapy for skeletal metastases showed superior results compared to the other regimens. Consumption of pain medication was notably lower in the 8Gy group compared to the 30Gy group. The 8Gy/single fraction demonstrated superiority to the other multiple dose patterns (20Gy and 30Gy) in controlling pain. Pathological fractures and spinal cord compressions occurred significantly less often in the 8Gy group compared to 20Gy and 30Gy groups, along with a reduce rate of re-irradiation. Furthermore, the 8Gy group reported the lowest rate of non-response among patients. No differences in survivorship among the dose patterns was detected.
